# Evolution of acaricide resistance of *Rhipicephalus decoloratus* on commercial farms in South Africa

**DOI:** 10.1007/s10493-023-00820-4

**Published:** 2023-07-08

**Authors:** Ellie M. S. P. van Dalen, Candice Jansen van Rensburg

**Affiliations:** Department of Zoology & Entomology, University of the Free State, PO Box 339, Free State Bloemfontein, South Africa

**Keywords:** Amitraz, Cypermethrin, Chlorfenvinphos, *Rhipicephalus decoloratus*, Resistance, Commercial farms, Evolution

## Abstract

The development of tick resistance to chemical control plays a major role in the increasing global economic impact of ticks on cattle farming. Reports on acaricide resistance of *Rhipicephalus decoloratus*, endemic to Africa and South Africa, are relatively few compared to the closely related and globally distributed *Rhipicephalus microplus*. In South Africa, ectoparasite control became the sole responsibility of each commercial producer when compulsory dipping was phased out from 1984. Different acaricidal management strategies resulted in the simultaneous development of resistance to various acaricide groups. The establishment of a Pesticide Resistance Testing Facility provided the opportunity to test *Rhipicephalus* (*Boophilus*) populations, submitted from all over South Africa, for resistance where failure of chemical control was experienced. The number of populations resistant to cypermethrin (CM) was significantly higher than those tested as resistant to amitraz (AM), or chlorfenvinphos (CFVP). No significant difference was found between the number of populations resistant to AM and CFVP. The evolution of *R. decoloratus* resistance at the end of a 12 year period indicated a stable but high prevalence of 90% overall resistance to CM. The same trend was seen for AM-resistant *R. decoloratus* populations but at a lower level of just over 40%. In contrast, CFVP resistant *R. decoloratus* populations showed a decreasing trend with near-total reversion to susceptibility. Multi-resistance was present in more than 50% of populations tested with the highest incidence in the Eastern Cape, KwaZulu-Natal, and Western Cape provinces.

## Introduction

Tick control stems from the tick’s ability to influence its host negatively and acting as a vector of pathogenic species such as *Anaplasma*, *Borrelia*, and *Babesia* (Jongejan and Uilenberg 2004; de la Fuente et al. 2008; Matysiak et al. 2016). Physical damage to the host itself in the form of anaemia, and damage to hides and teats (Hurtado and Giraldo-Rios 2018) is triggered by the tick’s hematophagous lifestyle. These conditions cause production losses in the form of lowered meat and milk production and further contribute to the economic impact (Jonsson et al. 1998) of high tick infestations. These adverse effects decrease the value of the end product, endanger food security, and necessitate the use of control, most frequently in the form of acaricides. The development of tick resistance to the major chemical classes of these acaricides (Rajput et al. 2006; Li et al. 2007; De La Fuente et al. 2008; Rodríguez-Vivas et al. 2011) is one of the most debilitating factors in the control of ticks, causing an increased financial burden on the already high economic impact of tick control. Although figures on current economic losses in South Africa are unavailable, it was estimated to be around R70 million per annum some 40 years ago when mortality losses, acaricide control, and vaccine costs were considered (Van Rensburg 1981). In 1985, the total losses in South Africa were placed at R550 million by a panel of the Food and Agriculture Organisation (Spickett et al. 2011). Today, almost 40 years later, an extrapolated estimation can be as much as R670 million if only the cattle ecto-parasiticides sold is considered (pers. comm. with industry agent).

*Rhipicephalus microplus*, a globally found one-host tick species, has been well studied, especially for its development of resistance to chemical control (Abbas et al. 2014). The African blue tick, *R. decoloratus*, endemic to Africa south of the Sahara and in South Africa in tropical and subtropical environments (Walker et al. 2003), is closely related to *R. microplus.* It is a one-host tick that prefers cattle as the main host but can also feed on wild ungulates in the absence of cattle (Horak et al. 2018). Field data on the development of resistance of *R. decoloratus* in Africa and South Africa is relatively limited and still needs to be extensively investigated.

In South Africa, the resistance of *R. decoloratus* to most classes of acaricides, was sporadically reported over the years, starting with arsenic in 1937, cyclodiene and toxaphene in 1948, DDT in 1954, organophosphorus and carbamate in 1966, pyrethroids in 1987, and formamidines in 1997 (George et al. 2004). Baker et al. (1978) found organophosphate resistance of *R. decoloratus* populations collected from communal areas in the eastern parts of the Eastern Cape Province, followed by reports of two field populations susceptible to amitraz (AM) and organophosphates (OP) tested at Kwanyanga Research Station near East London nearly 10 years later (Coetzee et al. 1987). Coetzee et al. (1987), however, found the two strains to be resistant to pyrethroids, including cypermethrin (CM). Mekonnen et al. (2002, 2003) later indicated resistance to AM, CM, and chlorfenvinphos (CFVP) on commercial farms in the Eastern Cape and North West provinces. A National Tick Resistance Survey (NTRS), conducted from 1997 to 2001, determined the resistance status of randomly collected *R. decoloratus* field populations to AM, CM, and CFVP, collected from all provinces in South Africa. This survey indicated resistant populations for all three acaricides, especially in areas along the coastline of South Africa, where favourable conditions such as high humidity and temperature are prevalent throughout the year (Van Dalen and Jansen van Rensburg 2023).

Resistance development may be caused by many factors, of which the frequency of use of a specific acaricide is the most important (Mekonnen et al. 2002; Abbas et al. 2014). Increased frequency of use usually takes place as a response to loss of efficacy causing emerging resistance to be both a cause and consequence of emerging resistance (NN Jonsson, pers. comm.). In South Africa, compulsory dipping for tick control, phased out from 1984 (Government gazette 1984), had the consequence that ectoparasite control on commercial farms became the sole responsibility of each producer. From then onwards, each producer followed his management strategy that often differed from the neighbouring farm in terms of the type of acaricide used. The availability of many different tick control remedies on the market also led to confusion on which remedy to choose and cost implications mainly were the primary consideration in the final decision.

In this confusion, a remedy with an active ingredient, similar to that previously used, could have been purchased, with the result that tick resistance development to that active ingredient was further promoted. The short generation time of *R. decoloratus* makes it possible for this tick species to complete 3–4 generations within 1 year when favourable conditions and abundant host availability are present (Pegram et al. 1986). Intervals of 7 days between acaricide exposures can theoretically lead to between nine to 12 exposures per year. This makes the rapid development of tick resistance to the acaricide in use inevitable within a short period. Climate change also caused winter dipping to become more regular, especially close to the coastal areas, with a consequently higher exposure of one-host ticks to acaricides, and an increase in resistance development.

The presence of resistant individuals in a population is not static and many factors can contribute to the development and possible disappearance of resistance. This study aimed to report on the results of phenotypic resistance profiles of *R. decoloratus* populations obtained from commercial farms in South Africa from 2006 to 2017. Three objectives were set: (1) to obtain a more recent and inclusive indication of total acaricide resistance development of *R. decoloratus* populations in South Africa, (2) to elucidate a possible development of *R. decoloratus* resistance over 12 years, (3) to evaluate the evolution of multi-resistant populations for the various provinces. Lessons learned can contribute to the prevention of uncontrolled development of tick resistance and extend the usable lifetime of currently available acaricides for tick control.

## Materials and methods

### Study material

South Africa is divided into nine provinces and 52 districts representing 213 local municipalities and covers a land area of 1 220 813 km^2^ (Municipalities of South Africa 2019). Eight provinces are totally or partially suitable for *R. decoloratus* to survive as these areas have temperate climatic conditions and cattle pastures that include grasslands and wooded areas (Walker et al. 2003). A mean annual number of 13 822 428 (13 813 700–14 313 935) cattle distributed over the eight provinces were available as the main host for *Rhipicephalus* species from 2006 to 2017 (DAFF 2019).

Cattle producers and pharmaceutical companies were encouraged to collect tick populations from commercial farms where indications or perceptions of tick resistance to acaricides used, were found. Communal farming practices also found in South Africa were excluded from this study, as they do not represent closed farming systems with individual control practices.

### Experimental procedure

Tick populations collected from 2006 to 2017 were evaluated at the PRTF, located in the Department of Zoology and Entomology at the University of the Free State in Bloemfontein, South Africa. The larval immersion test (LIT), developed by Shaw (1966) and employed to evaluate *R. decoloratus* population resistance to AM, CM and CFVP, produced a phenotypic resistance profile for each population tested. These acaricides and testing methodology were chosen due to a history of previous use in South Africa (Coetzee et al. 1987; Mekonnen et al. 2002; Ntondini et al. 2008). The recommended field concentration on each dip acaricide was considered the discriminating concentration (DD) to which efficacy needs to be determined. This assumption was derived from the conjecture that efficacy for any registered acaricide remedy, when applied in the field, should be at least 90–100%. Tick populations exposed to field concentrations of these acaricides should, therefore, at least fall into this category to be considered susceptible.

Upon receipt of the field strain, an appropriate identification number was allocated to the collection. Engorged female ticks were identified as either *R. decoloratus* or *R. microplus* by using the dentition differences between the two blue tick species (Walker et al. 2003). Engorged females, identified as *R. decoloratus* and destined for LIT, were transferred to Erlenmeyer flasks (ca. 20 ticks per flask). The flasks were then incubated at > 75% RH in an environmental room kept at 25–28 °C to allow oviposition and hatching of the larvae. The date of the hatch was determined as the date when approximately 75% of larvae had hatched. Three commercially available acaricide classes were used, CM found in Curatik (15% m/vol, 2006–2008) and Pro-dip (20% m/vol, 2008–2017), AM found in Triatix 125 (12.5% m/vol, 2006–2017) and CFVP found in Disnis NF dip (9% m/vol, 2006–2008) and Coopers Supadip (30% m/vol, 2009–2017). Due to 12 years elapsing from the first to the last test conducted, different batches of each acaricide had to be used (Table 1).

**Table 1:** Acaricides used during the 12 years of tick resistance testing at the pesticide resistance testing facility, University of the Free State, Bloemfontein, South Africa

Remedy name		Batch	Manufacturer	Concentration (% m/vol)	Period used	Expiry date
Cypermethrin	Curatik	22117196	Virbac	15	2006–2007	February 2007
	Pro-dip	R1092		20	2008–2009	January 2013
		P2645			2010–2013	July 2013
		W1004			2014–2017	August 2017
		W 1284			2015–2017	December 2017
Amitraz	Triatix 125	605 008	Afrivet	12.5	2006–2008	April 2008
		811 027			2008–2010	October 2010
		006 006			2010–2012	December 2012
		004 05 A			2013–2014	December 2014
		3098			2015–2017	August 2017
Chlorfenvinphos	Disnis NF dip	22120208EC04	Bayer	9	2006–2008	December 2008
	Coopers supadip	04612-03	Afrivet	30	2009–2010	November 2010
		00204 A			2010–2013	May 2013
		00298 A			2013–2013	November 2013
		00487 A			2013–2015	May 2015
		00697 A			2016–2017	April 2017

*Rhipicephalus decoloratus* larvae were exposed to the field concentration recommended for each remedy to be effective for tick control of susceptible tick species. Exposure took place 16–21 days after hatching of the larvae. An initial 1% stock solution was prepared in double distilled water to obtain dilutions of the field concentrations of 0.025% for amidines, 0.015% for pyrethroids, and 0.05% for OPs. Double distilled water was used as a control solution. The methodology of exposure, as initially described by Shaw (1966) and in more detail for use at the PRTF (Van Dalen and Jansen van Rensburg 2023), was followed. In short, approximately 200 larvae were exposed in a filter paper sandwich to 10 ml of each test solution for 10 min. After exposure, approximately 100 larvae were transferred to two replicates of dry filter paper envelopes, closed off and incubated at the same conditions as for oviposition and hatching. After 72 h of incubation, the numbers of live vs. dead larvae were determined and documented, and the efficacy of the acaricide was calculated. Larvae from each suitable population received were exposed to all three acaricides for multi-resistance detection, even when testing for resistance of only the acaricide in use was requested.

### Data analysis

Abbott’s formula was applied to determine corrected mortalities for each population. For this formula, the mean of the duplicate tests, compared to the mean of the control sample, allowed for corrections due to incidental mortalities (Abbott 1987). Assays with control values of > 10% were either repeated or discarded. Results for a population were interpreted as an indication of resistance if the mortality was 0–49%, emerging resistant if mortality was 50–79%, and susceptible if 80–100%. Although the category between 80 and 89% was considered effective with reservation it was included in the susceptible range to allow for more leniency towards susceptibility. The significance of the difference in acaricide resistance between the three acaricides was tested with a two-sample *t *test assuming unequal variance (α = 0.05). Resistance trends followed and *t *test statistics were analysed by making use of Microsoft Excel.

### Safety measurements

All ticks and larvae were contained and tested in a Section 20 accredited laboratory, adhering to good laboratory practices. A dedicated room, separate from the chemical mixing room, was used for tick resistance testing to prevent contamination with the concentrated remedies.

After completing the assays, remaining adult ticks, eggs and larvae were destroyed by treatment with acetone, 70% alcohol, followed by heat treatment through submersion into boiling water. The dead ticks and larvae, filtered through a funnel with filter paper, were discarded into a biological waste container, picked up periodically for incineration by an accredited waste disposal service.

## Results

### Blue tick collections

**Fig. 1:**
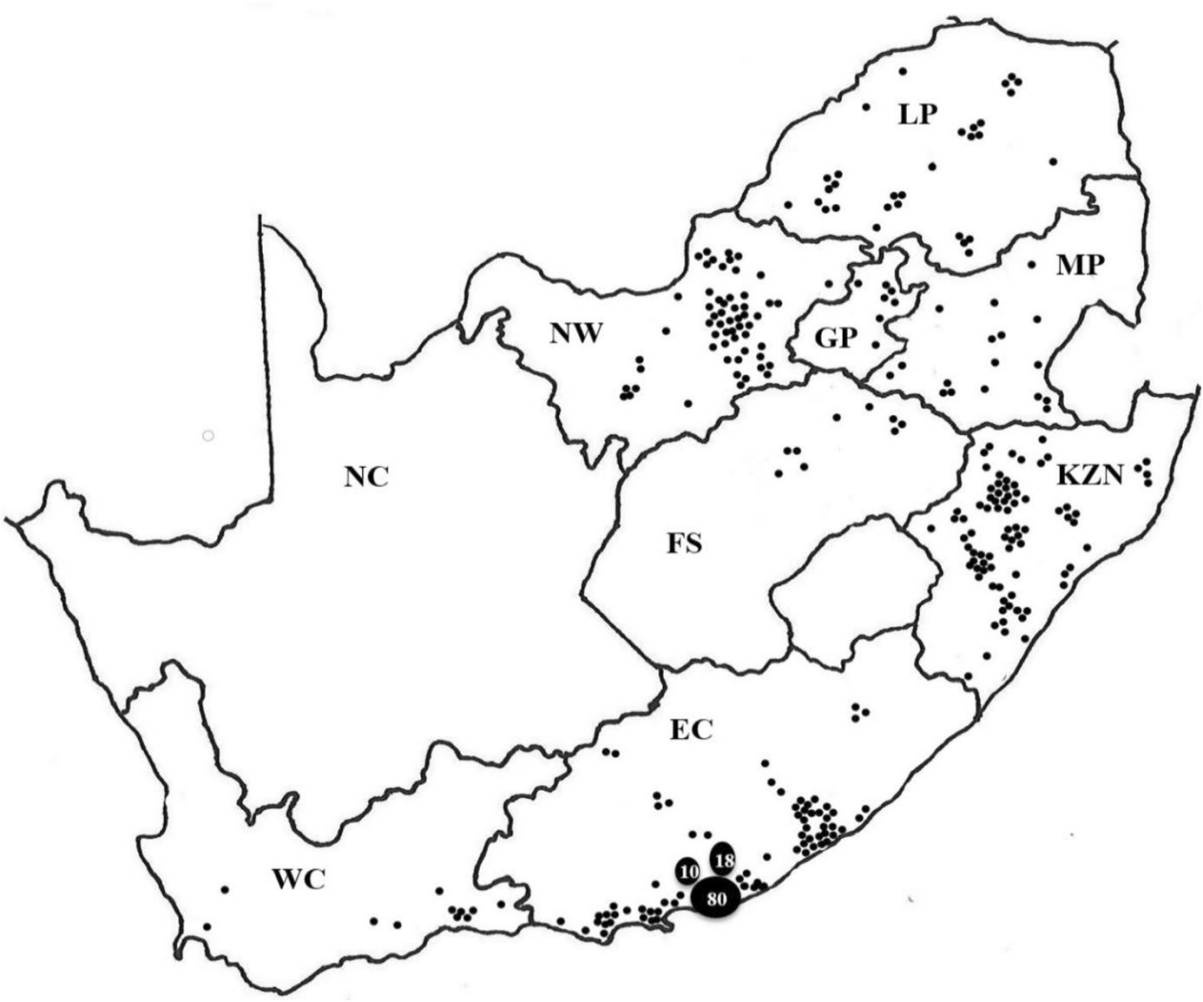
Collection points of field populations of *Rhipicephalus* *decoloratus* (●) in the different provinces in South Africa from 2006 to 2017. Each dot represents one population, except for the Eastern Cape Province where the number of populations is indicated by a number for the three districts as too many populations were received to be indicated by single dots alone. Provinces: *FS* Free State, *KZN* KwaZulu-Natal, *MP* Mpumalanga, *LP* Limpopo, *EC* Eastern Cape, *NW *North West, *GP* Gauteng, *WC* Western Cape, *NC* Northern Cape

*Rhipicephalus decoloratus* populations were submitted from 369 commercial farms during a period of 12 years from 2006 to 2017. Tick collections that were received were mostly from areas along the south–eastern to eastern coastal regions of South Africa and then inland towards the north–west (Fig. 1). The highest number of tick populations were submitted from the Eastern Cape Province with 155 populations received, followed by KwaZulu-Natal (80), North West (58), Limpopo (30), Mpumalanga (17), the Western Cape (13), nine from the eastern parts of the Free State and seven from Gauteng provinces (Table 2).

**Table 2:** A summary of the resistance status of *Rhipicephalus decoloratus *populations found to be susceptible (S), emerging resistant (ER), and resistant (R) to cypermethrin, amitraz and chlorfenvinphos for each province in South Africa. The number of populations received is also expressed as a percentage of the populations received from each province in parenthesis

Province	No. populations received				Resistance profile for each province					
		Cypermethrin (150 ppm)			Amitraz (250 ppm)			Chlorfenvinphos (500 ppm)		
		S	ER	R	S	ER	R	S	ER	R
Eastern cape	155	9 (5.8)	17 (11.0)	129 (83.2)	58 (37.4)	45 (29.0)	52 (33.5)	106 (68.4)	26 (16.8)	23 (14.8)
KwaZulu-Natal	80	7 (8.8)	9 (11.3)	64 (80.0)	39 (48.8)	21 (26.3)	20 (25.0)	57 (71.3)	15 (18.8)	8 (10.0)
North west	58	7 (12.1)	12 (20.7)	39 (67.2)	50 (86.2)	4 (6.9)	4 (6.9)	55 (94.8)	3 (5.2)	0
Limpopo	30	4 (13.3)	7 (23.3)	19 (63.3)	23 (76.7)	3 (10.0)	4 (13.3)	27 (90.0)	2 (6.7)	1 (3.3)
Mpumalanga	17	4 (23.5)	6 (35.3)	7 (41.2)	14 (82.4)	1 (5.9)	2 (11.8)	16 (94.1)	1 (5.9)	0
Western cape	13	3 (23.1)	2 (15.4)	8 (61.5)	8 (61.5)	3 (23.1)	2 (15.4)	10 (76.9)	1 (7.7)	2 (15.4)
Free State	9	2 (22.2)	2 (22.2)	5 (55.6)	8 (88.9)	1 (11.1)	0	9 (100)	0	0
Gauteng	7	0	4 (57.1)	3 (42.9)	6 (85.7)	0	1 (14.3)	7 (100)	0	0
Total	369	36 (9.8)	59 (16.0)	274 (74.3)	206 (55.8)	78 (21.1)	85 (23.0)	287 (77.8)	48 (13.0)	34 (9.2)

### Acaricide resistance profile: *Rhipicephalus decoloratus*

Severe resistance of *R. decoloratus* populations exposed to the field concentration of CM was found throughout South Africa. When the mean percentage resistance for each province was compared in a two-sample *t *test assuming unequal variance, CM resistance was found to be higher than resistance to AM (t = − 9.4, d.f. = 6, P = 2.8 × 10^−6^) and CFVP (t = − 14.0, d.f. = 11, P = 2.8 × 10^−8^). No significant difference was found between resistance to AM and CFVP (t = 1.81, d.f. = 11, P = 0.097).

**Fig. 2:**
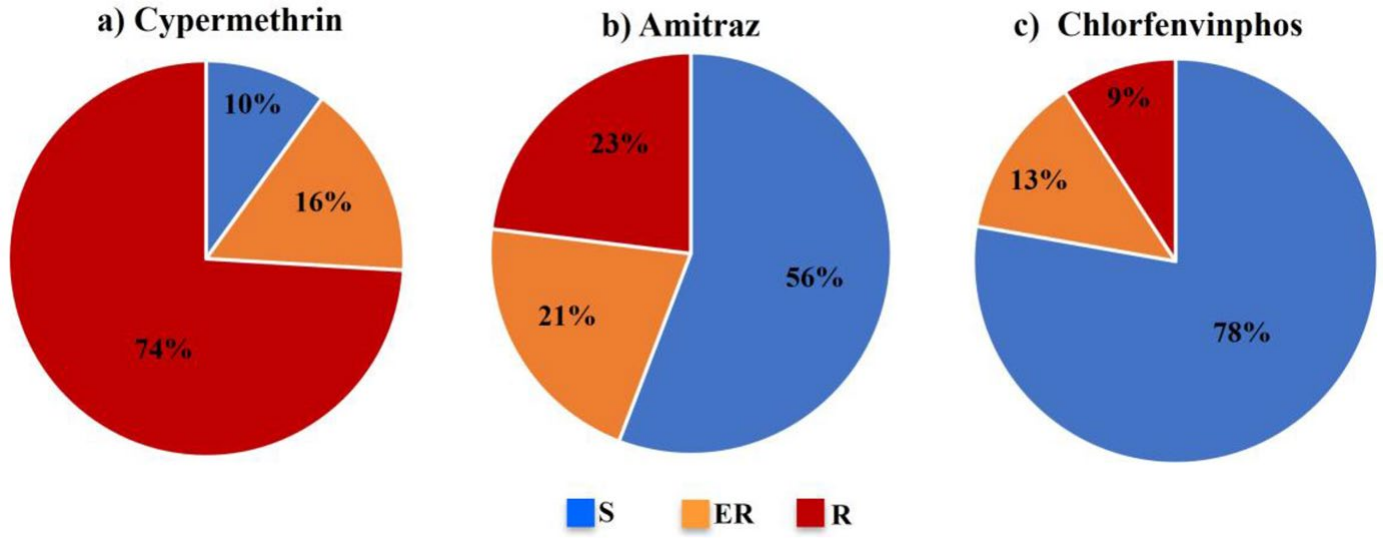
A summary of the resistance profile of *Rhipicephalus* *decoloratus* populations collected from 2006 to 2017 in South Africa for **a** cypermethrin, **b** amitraz, and **c** chlorfenvinphos, expressed as percentage received of the total (*S* susceptible, *ER* emerging resistant, *R* resistant)

Exposure to CM at a concentration of 150 ppm showed 74.3% (274) of the 369 populations to be resistant, 16.0% (59) to be emerging resistant and only 9.8% (30) of the populations were still susceptible to CM (Table 2; Fig. 2a). There was also a high prevalence of resistance to CM on a provincial level. The Eastern Cape Province showed the highest prevalence with 83.2% of the populations received from this province that tested as resistant, followed by KwaZulu-Natal with 80.0%, North West with 67.2%, Limpopo with 63.3%, Western Cape with 61.5%, Free State with 55.6%, Gauteng with 42.9% and Mpumalanga with 41.2% (Table 2).

Populations tested as resistant when exposed to 250 ppm AM were found in 23.0% (85) of the total number of populations tested. A further 21.1% (78) of the populations displayed emerging resistance to AM and 55.8% (206) tested as susceptible (Table 2; Fig. 2b). The province with the highest percentage AM resistant populations was Eastern Cape with 34%, followed by KwaZulu-Natal (25%), Western Cape (15%), Gauteng (14%), Limpopo (13%), Mpumalanga (12%) and North West (7%). Free State Province had no AM resistant populations (Table 2).

The total number of populations resistant to CFVP, when exposed to a concentration of 500 ppm, was only 9.2% (34) of the total number of 369 populations tested. Emerging resistance was found in a further 13.0% (48) with 77.8% (287) of the populations classified as susceptible to CFVP (Table 1; Fig. 2c). Four provinces produced populations resistant to CFVP. Eastern Cape and Western Cape showed 15% of the populations to be resistant to CFVP followed by KwaZulu-Natal (10%) and Limpopo (3%) (Table 2).

### Evolution of resistance over 12 years

**Fig. 3:**
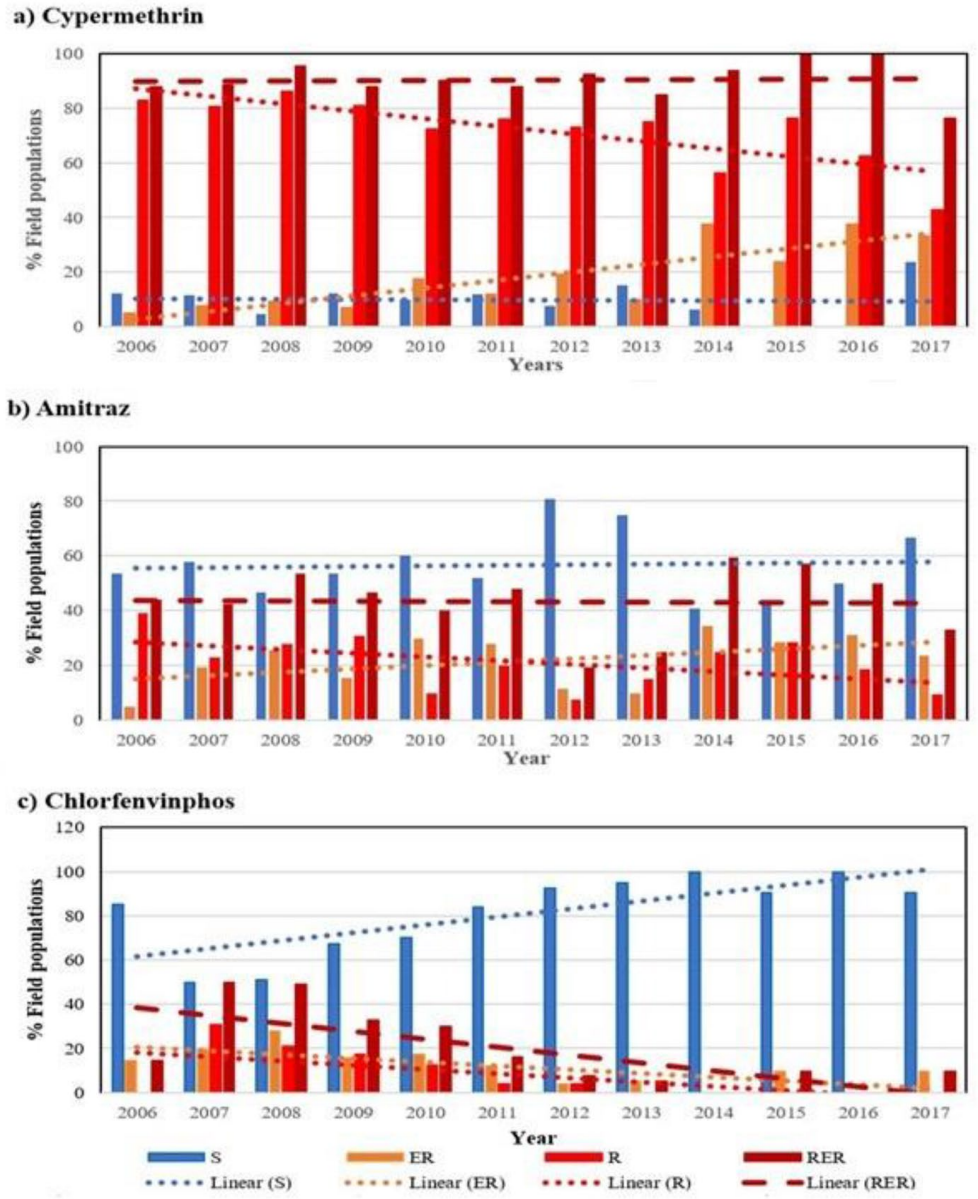
The total number (percentage) of *Rhipicephalus* *decoloratus* populations tested per year for 12 years in South Africa indicated as susceptible (S, blue bars), emergent resistant (ER, orange bars), resistant (R, red bars) and the combination of emerging resistant and resistant populations (RER, dark red bars) to **a** cypermethrin, **b** amitraz, and **c** chlorfenvinphos. The linear trend lines for each aacaricide and resistance profile, are indicated by the dotted lines. (Color figure online)

A year-to-year evaluation of the data showed the evolution of tick resistance to all three acaricides tested from 2006 to 2017 (Fig. 3). Linear trend lines for CM showed a decrease in the percentage of populations tested as resistant over this period (Fig. 3a). Despite this decrease, populations received in 2017 still indicated the presence of > 40% CM resistant populations. The downward trend of populations resistant to CM was accompanied by an increase in the trend of populations tested as emerging resistant (Fig. 3a). When resistant and emerging resistant populations were combined to indicate a < 80% efficacy for each population, the trend line from this summation showed a stable line over the 12 years. The percentage of populations tested as susceptible to CM was also stable over the evaluation period.

Linear trend lines for the percentage populations resistant to AM showed a gradual decrease in resistance with an increase in the emerging resistant populations over the 12 year period (Fig. 3b). The trend line for susceptible populations showed no significant increase or decrease over this period (Fig. 3b). Trend lines for the summation of resistant and emerging resistant populations were also stable over the evaluation period.

In contrast to CM and AM resistant populations, both the percentage of the populations resistant and emerging resistant to CFVP showed a steady decline from 2006 to 2017, as indicated by the linear trend lines (Fig. 3c). In this case, the summation of resistant and emerging resistant populations also showed a downwards trend close to 0% for 2017. The percentage of susceptible populations, when exposed to CFVP, showed an increase of 20% over the 12 years. From 2011 onwards, > 80% of populations tested per year were susceptible to CFVP (Fig. 3c).

### Multi resistance to acaricides

**Fig. 4:**
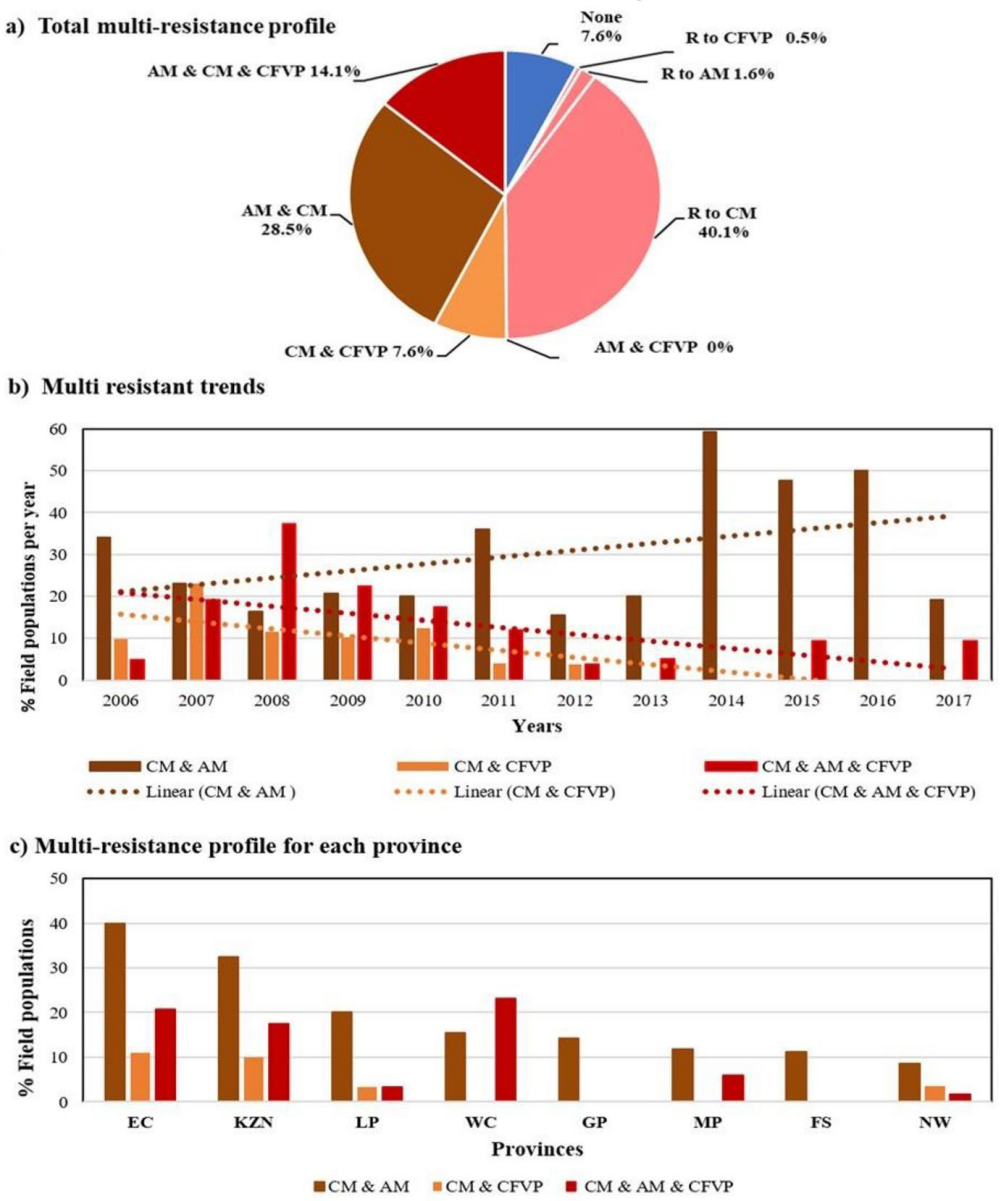
A summary of **a** the resistant (R) and multi-resistance profiles of all *Rhipicephalus decoloratus* populations collected; **b** the multi-resistant populations to acaricide groups for each year (bars) and overall resistant trends of *R. decoloratus* populations to combinations of acaricides over 12 years (dotted lines); and **c** the multi-resistance profile for each province, from 2006 to 2017 in South Africa. Acaricides investigated were amitraz (AM), cypermethrin (CM), and chlorfenvinphos (CFVP). Provinces are indicated in the figure as Eastern Cape (EC), KwaZulu-Natal (KZN), Limpopo (LP), Western Cape (WC), Gauteng (GP), Mpumalanga (MP), Free State (FS) and North West (NW)

Populations were considered multi-resistant when < 80% efficacy was obtained for exposure to more than one acaricide. Only 7.6% (28) of the 369 populations received were still susceptible to all three acaricides tested. Populations that were only resistant or emerging resistant to CM were found in 40.1% (148) of the populations tested, to only AM in 1.6% (6) and to only CFVP in 0.5% (2) of the populations (Fig. 4a).

Populations resistant or emerging resistant to two acaricides were found in 36% (133) of the populations tested, 28.5% (105) to both CM and AM, and 7.6% (28) to both CM and CFVP (Fig. 4a). No populations were shown to be resistant or emerging resistant to both AM and CFVP. Populations resistant or emerging resistant to all three acaricides were found for 14.1% (52) of populations tested (Fig. 4a).

Multi-resistance trends from 2006 to 2017, however, showed that there was a decreasing tendency in populations resistant or emerging resistant to all three acaricides tested (Fig. 4b). Populations resistant or emerging resistant to both CM and CFVP also showed a downwards trend, but those resistant or emerging resistant to both CM and AM had an upwards trend (Fig. 4b).

All provinces had populations that tested as either resistant or emerging resistant to the combination of CM and AM (Fig. 4c). The highest frequencies were found in Eastern Cape (40%) and KwaZulu-Natal (33%) followed by Limpopo (20%), Western Cape (15%), Gauteng (14%), Mpumalanga (12%), Free State (11%) and North West (9%). The number of samples received from the Western Cape, Free State, and Gauteng provinces were low and may not be an accurate representation of multi-resistance. Four provinces produced populations resistant or emerging resistant to the combination of CM and CFVP: Eastern Cape (11%), KwaZulu-Natal (10%), Limpopo (3%), and North West (3%). Multi resistance of populations either resistant or emerging resistant to all three acaricides were found in Eastern Cape (21%), KwaZulu-Natal (18%), Limpopo (3%), Western Cape (23%), Mpumalanga (6%), and North West (2%) (Fig. 4c).

## Discussion

### Overall acaricide resistance

Field populations of *R. decoloratus*, collected in South Africa during a NTRS between 1998 and 2001, showed the presence of resistance to all three acaricide groups tested. Although only 3.3% of the populations in the survey had resistance to AM, 29.4% were resistant to CM and 20.0% to CFVP (Van Dalen and Jansen van Rensburg 2023). For populations not chosen explicitly due to resistance problems experienced, these results already indicated previous high use of CM and CFVP for tick control during the time preceding this survey.

In the current study, tick populations submitted by pharmaceutical companies mainly were from clients experiencing problems with tick control. This made results more biased towards areas where resistance development to the acaricide in use might already have been established. It must also be kept in mind that changes in control practices may cause increased or decreased resistance scenarios on each farm as specific farms were not followed to monitor the resistance development over time. The 12 year review of resistance profiles from populations submitted from different commercial farms is, however, useful to highlight general aspects of tick resistance development and to indicate tendencies of use of the various acaricide groups on commercial farms in South Africa over time.

Summarised results of field populations of *R. decoloratus* tested from 2006 to 2017, showed a significantly higher incidence of populations resistant to CM (74%) than to AM (23%). In contrast, a lower incidence of 9.2% of populations resistant to CFVP was observed. When the resistance trends over 12 years were evaluated, a decrease in the percentage of populations resistant and an increase in emerging resistant populations was found for CM and AM. The summation of these two values to specify resistance in terms of < 80% sufficient control was constant for this time frame for both acaricides. These results may indicate the fluctuation of resistant populations reverting to susceptibility and susceptible populations progressing towards resistance as a result of different acaricide management strategies followed on each commercial farm over time. Of concern was the high resistance trend that occurred for CM at around 90%, followed by AM at about 55%, over the 12 years of review. Further monitoring of these trends will be important for resistance management planning. Petermann et al. (2016) found that for the closely related *R. microplus* studied in New Caledonia, populations resistant to AM increased from 0 to 60% in 12 years when treated every 3–4 weeks. In Mexico (state of Veracruz), 90.6% *R. microplus* strains were resistant to pyrethroids after 15 years in use (Fernández-Salas et al. 2012); and 86.4% of populations were resistant to CM after 20 years of use in the state of Sao Paulo in Brazil (Mendes et al. 2011). Petermann et al. (2016) further only found limited reversion of deltamethrin resistant *R. microplus* populations in New Caledonia, 11 years after cessation of use.

Results of the current study displayed similar resistance development patterns for *R. decoloratus* to CM and AM as was found by Petermann et al. (2016) for *R. microplus.* The increase in the percentage resistant or emerging resistant populations during a NTRS done at the turn of the century (Van Dalen and Jansen van Rensburg 2023) up to the current study was from 35.5 to 90% for CM, and from 6.6 to 44.1% for AM. However, in South Africa, contrary to New Caledonia, there have been no state-organized initiatives to coordinate the use of acaricides since 1984 (Animal Disease Act No 35). Implementation of ectoparasite control, control management strategies, acaricides used and frequency of treatments are done at the sole discretion of each producer (Rodríguez-Hidalgo et al. 2017) and can differ from farm to farm. This has caused the acaricide resistance profile of tick populations to be specific for each farm. Many producers lack critical knowledge on the chemical composition of different remedy brands on the market, making cost implications the main determining factor for purchase.

These decisions can sometimes aggravate resistance to a specific acaricide when an alternative brand name containing the same acaricide group is purchased. Indiscriminate increase and decrease of application concentrations and use of homebrew mixtures by producers also promotes the possibility of resistance development (Vudriko et al. 2018). An unreliable history of acaricide use with no information on the start of use up to confirmed resistance to any acaricide, makes it difficult to determine an exact timeline for resistance development of *R. decoloratus* to AM or CM on commercial farms in South Africa. The high prevalence of resistance to pyrethroids can also be due to unintentional exposure of ticks to pyrethroids that control other ectoparasites, such as flies, thus keeping tick resistance levels high (Jonsson et al. 2000). Rodríguez-Vivas et al. (2011) reported that resistance to pyrethroids is gained faster than its reversion to susceptibility after decreased use. Use of pyrethroids in South Africa in alternation or rotation with other acaricides for control of *R. decoloratus* might have been a further factor that caused a stepwise augmentation of populations resistant to pyrethroids (Rodríguez-Vivas et al. 2011). This might explain the consistently high levels of resistant pyrethroid populations found in the current study.

On communal farms, dipping compounds are purchased by tender by the state authorities with longer periods of use implemented and without intermittent use of other acaricides. This practice could potentially lead to the selection for acaricide resistance, but Ntondini et al. (2008) found a low incidence of AM resistance in a communal area where it has been used for several years on state tender.

In contrast, the presence of CFVP resistant *R. decoloratus* populations showed a downward trend for both resistant and emerging resistant populations and an upward trend for populations susceptible to CFVP over the 12 year review. Shaw (1966) first reported resistance of ticks to OPs in South Africa in 1966. Coetzee et al. (1987) showed total susceptibility of *R. decoloratus* to OPs in the Eastern Cape Province in 1987. During the NTRS from 1997 to 2001, 20.0 and 16.1% populations tested as resistant and emerging resistant to CFVP, respectively, although, a low use of OP remedies was indicated (Van Dalen and Jansen van Rensburg 2023). Mekonnen et al. (2002, 2003) also reported *R. decoloratus* populations resistant to CFVP, both on commercial and communal farming areas in the eastern parts of the Eastern Cape Province where OPs were not used during the 10 years preceding their study. In the present study, the decrease of *R. decoloratus* resistance to CFVP might indicate lower use of OPs by producers even before the start of the present study, accompanied by a slow reversion of *R. decoloratus* back to susceptibility for OPs. The use of OPs in South Africa was greatly discouraged due to its high toxicity for oxpeckers (Endangered Wildlife Trust 2009) and may have contributed to the lowered resistance of *R. decoloratus* to CFVP. Stone (1972) stated that the resistance status of ticks to OPs might be maintained in the complete absence of chemical treatment. The current study showed that a reversion of OP resistant *R. decoloratus* populations back to susceptibility might occur. However, the exact timeframe seems to be extended and can be prolonged to > 20–30 years of no use.

### Multi resistance

Multi-resistant populations were found in > 50% of *R. decoloratus* populations submitted, with populations resistant to all three acaricide groups tested evident in 14.1% thereof. Once again, resistance to CM was prevalent in all multi-resistant populations, with 28.5% of the tested populations resistant to both CM and AM and 7.6% resistant to CM and CFVP. No populations were found to be resistant to both AM and CFVP. This is in contrast to the results obtained from the NTRS conducted in South Africa at the turn of the century, where the main multi-resistant combination was found to be resistance to CM and CFVP (Van Dalen and Jansen van Rensburg 2023). A downward trend for multi-resistant populations was found over the 12 year period except for populations resistant to both CM and AM, where an upwards trend was seen. These results concluded that the use of remedies containing amidines increased since the previous survey as also confirmed by the steep increase of amidine resistance compared to results from the NTRS (Van Dalen and Jansen van Rensburg 2023). If so, the higher use of amidine-containing remedies could be contributing to the increased inefficacy of pyrethroid use. Remedies containing amidines and or pyrethroid may also have been the most promoted remedies in South Africa during this period. The downward trend of populations resistant to any acaricide combination with CFVP also confirms lower use of OPs since previous studies.

Multi-resistance to CM and AM was found in populations from all the provinces in South Africa with the highest incidence in the Eastern Cape, KwaZulu-Natal, and Western Cape provinces. These provinces are highly favourable for tick survival due to high rainfall, temperate climatic conditions, and favourable grazing conditions for the cattle hosts in the form of open grasslands (Walker et al. 2003; Horak et al. 2018). The high abundance of ticks would also involve more frequent acaricide treatments and may explain the occurrence of high acaricide resistance in these areas.

These results showed that the fight against tick resistance development to chemical control should be approached more holistically to both conserve the available acaricides for future use and to move away from only relying on chemical control. More recent tick control measures such as the use of growth regulators and tick-resistant cattle can be considered, although resistance of *R. microplus* was already been reported for growth regulators (Reck et al. 2014). In this study the use of ivermectin products were not evaluated in association with the other three acaricides tested but are also used indiscriminately by many commercial producers. Preliminary studies on three farms in the Eastern Cape province showed a pre-clinical occurrence of ivermectin resistance (unpubl. results). An overall incidence of ivermectin resistance in South Africa should also be investigated.

## Conclusion

The evolution of *R. decoloratus* resistance over 12 years showed a stable but high prevalence of overall resistance to CM. The same trend was seen for AM-resistant *R. decoloratus* populations but at a lower level. In contrast, CFVP-resistant *R. decoloratus* populations showed a decreasing trend with near-total reversion back to susceptibility at the end of the 12 year period. Multi-resistant populations were present in > 50% of *R. decoloratus* populations tested, with a downwards trend in all combinations, except for CM and AM. The highest incidence of multi-resistant populations was found in the Eastern Cape, KwaZulu-Natal, and Western Cape provinces. Acaricide control and resistance management strategies for the future should be more focused on determination of initial resistance presence on individual farms before recommendations for a specific acaricide to use is made especially for the provinces with high prevalence of multi-resistant populations. A more integrated management strategy, that include alternative acaricides such as growth regulators and non-acaricide strategies should also be considered to preserve chemical control remedies for prolonged use.

## Data Availability

The datasets generated during and/or analysed during the current study are available from the corresponding author on reasonable request.
